# Complete Genome Sequence of a Novel *Bacillus* sp. VT 712 Strain Isolated from the Duodenum of a Patient with Intestinal Cancer

**DOI:** 10.1128/genomeA.00786-16

**Published:** 2016-08-04

**Authors:** George Tetz, Victor Tetz

**Affiliations:** Human Microbiology Institute, New York, New York, USA

## Abstract

We report here the complete genome sequence of the spore-forming *Bacillus* sp. strain VT 712 isolated from the duodenum of a patient with intestinal cancer. The genome is 3,921,583 bp, with 37.9% G+C content. It contains 3,768 predicted protein-coding genes for multidrug resistance transporters, virulence factors, and daunorubicin resistance.

## GENOME ANNOUNCEMENT

Bacteria belonging to the genus *Bacillus* are phenotypically and genotypically heterogeneous; they are Gram-positive, spore-forming, rod-shaped, aerobic bacteria (1, 2). The sequence of the complete 16S rRNA gene of *Bacillus* sp. strain VT 712, which was isolated from the duodenum of a patient with intestinal cancer, was 98% identical to that of *Bacillus megaterium* DSM 319*,* a bacterium first described in 1884 as an environmental microorganism found in soil and not previously isolated from humans (3–5). Whole-genome paired-end sequencing of *Bacillus* sp. VT 712 was performed on an Illumina HiSeq sequencer and represented approximately 250-fold coverage of the genome (Illumina Co., USA). Overall, the draft genome contained 124 contigs that were assembled with the SPAdes genome assembler software, version 3.5.0 (6).

The genome, with 3,921,583 bp and 37.9% G+C content, was annotated using the NCBI Prokaryotic Genome Annotation Pipeline and Rapid Annotations using Subsystems Technology (RAST) (7, 8). The assembled genome contains 3,768 protein-coding genes and 163 predicted RNA genes, including 59 rRNAs, 99 tRNAs, and five noncoding RNA operons. The analysis indicated that the genome contains a significant number of antibiotic resistance genes, including those encoding resistance to vancomycin (*vanZ*, *vanB*, and *vanW*), tetracycline [*tet*(*A*)], and fosfomycin (*fosB*), as well as genes of multidrug resistance efflux pumps (ABC transporters, multidrug and toxin extrusion [MATE], major facilitator superfamily [MFS] transporters, and the multidrug transporter AcrB). The genome also encodes beta-lactamases and the quaternary ammonium compound resistance protein SugE, and it contains genes encoding proteins that confer resistance to tellurite, acriflavin, and organic hydroperoxide. BLAST analysis allowed the identification of genes encoding resistance to the chemotherapeutic drug daunorubicin, which was previously used in the patient (9). The genome also contained genes encoding virulence factors, such as hemolysin D, flagellar, and chemotaxis proteins, and numerous peptidases and ureases (10, 11).

Compared to the genome of *B. megaterium* strain DSM319 (GenBank accession no. NZ_CP010586.1), the phylogenetically closest member, *Bacillus* sp. VT 712, is smaller (5,097,447 bp versus 3,921,583 bp, respectively), has a higher G+C content (38.2% versus 37.9%, respectively), and has more protein-coding genes (5,272 genes versus 3,768 genes, respectively). An *in silico* DNA-DNA hybridization (DDH) analysis of the two genomes, using the Genome-to-Genome Distance Calculator (GGDC2.1) algorithm, produced a DDH value of 20.50%, which is well below the threshold value of 70% set for genomes belonging to the same species (12, 13).

The complete genome sequence of *Bacillus* sp. VT 712 will help determine the role of *Bacillus* species in human diseases, particularly in the cancer microbiome (14).

### Nucleotide sequence accession number.

The genome sequence from *Bacillus* sp. VT 712 is deposited in NCBI under the accession no. LWBK00000000.
